# The London Panel Committee

**Published:** 1915-06-26

**Authors:** 


### The London Panel Committee.

### HOSPITALS AND LOCAL PANEL ASSOCIATIONS.

The General Purposes Sub-Committee invited the London Panel Committee on June 22 to express its approval of the formation of local associations of practitioners on the panel. It was urged that such bodies would be of great value in dealing with problems peculiar to localities. In each area co-operative arrangements might be made for weekly and periodical holidays; rotas of medical referees might be appointed to advise in difficult or doubtful cases, in districts in which sickness incidence was heavy practitioners might encourage health lectures and work for sanitary reforms. Negotiations with hospitals and arrangements for specialist consultations might sometimes be better carried out by a local association.

The Committee passed a resolution expressing the opinion that practitioners on the panel in the various areas of London should be grouped under district panel associations, also that the Panel Committee should arrange local meetings of practitioners from time to time.

A statement was submitted showing that the number of practitioners on the panel resident in London was 1,550, of whom 457 were registered since 1900, and presumably were eligible for service with the Forces. The number of practitioners on the panel at present absent on military duty was 125. The General Purposes Sub-Committee remarked that it would appear that only about one-quarter of the eligible practitioners were actually serving with the Forces, and that the difficulty was a fear that on returning practitioners would find their practices much depleted by reason of the transference of insured persons to other lists. The Panel Committee passed a series of resolutions expressing the opinions:

(1) That the right of an insured person to transfer should be temporarily suspended.

(2) That practitioners on the panel should collectively arrange to release an adequate proportion of their number for service with the Forces.

(3) That in the event of a colleague accepting a commission the practitioners undertaking the treatment of the persons on the list should, in normal circumstances, permit the colleague to retain 50 per cent. of the income from the Insurance practice.

A detailed scheme embodying these resolutions was also approved.

